# Spatial influence on the distribution of downhill skiers in Sweden

**DOI:** 10.1007/s00484-022-02259-5

**Published:** 2022-03-31

**Authors:** Martin Falk, Eva Hagsten, Xiang Lin

**Affiliations:** 1 University of South-Eastern Norway, School of Business, Campus, Bø, Norway; 2 Södertörn University, Huddinge, Stockholm Sweden

**Keywords:** Snow depth, Ski resorts, Skier visits, Spatial econometric models, Sweden

## Abstract

This study investigates empirically how natural snow depth affects the number of downhill skiers. Data include the number of skier visits for the 32 largest ski resorts in Sweden from the 1998/1999 to the 2018/2019 seasons. Results of spatial dynamic estimations show that an increase in natural snow depth in the ski area has a significant negative impact on the number of skier visits in the short term, although the magnitude is small. This implies that a snow deficit leads to increased demand for downhill skiing both directly and indirectly (in the neighbouring areas). The variable snow depth in the neighbouring ski areas is not significantly different from zero, indicating that no spatial substitution takes place. There is, however, a strong positive relationship between skier visits to neighbouring areas, revealing that ski resorts are complements rather than substitutes. The long-term influence of snow depth is not significant, implying that the ski business is independent of variations in snow depth. Instead, the number of skier visits is mainly determined by past visits, revealing a high degree of persistence.

**Supplementary Information:**

The online version contains supplementary material available at 10.1007/s00484-022-02259-5.

## Introduction

In Sweden, there are about 10 million downhill skier visits per season, of which more than 80 per cent are accounted for by the 32 largest ski resorts (SLAO 2019). A large proportion of these resorts are located in the counties of Dalarna and Jämtland. As opposed to the development in many other countries, Sweden does not experience a decline in demand for downhill skiing, despite a receding snow depth in large parts of the country (Wern 2015, SLAO various years).

Recent projections indicate that the January to March snow depth in the Northern Hemisphere may decrease by more than 20 per cent by 2080–2100 (Zhu et al. 2021). The number of frost days in the county of Jämtland is expected to decrease by 35 days until 2050 compared with the mean for the years 1971–2000, based on the medium emission scenario RCP 4.5.^1^ This scenario also projects a decrease in the number of snow days with more than 5 mm of water content from 260 to 220, and 200 days based on the high emission scenario (RCP 8.5) in the snowy mountainous region in the western part of the county (Nylén et al. 2015). The lower eastern part of the area is expected to experience a change from 150 to 115 (RCP 4.5) or 80 days (RCP 8.5) (Nylén et al. 2015). Rice et al. (2021) find that the average length of the season in the northern ski areas of Sweden is significantly less shortened compared to those in central and southern Sweden under the high emissions scenarios at mid-century. Given this trend and future scenarios, knowledge on how skiers react to variations in natural snow depth is of interest to both stakeholders and researchers.

The aim of this study is to empirically investigate how variations in natural snow depth affect the number of downhill skiers. Based on an empirical model, both the persistence of skier demand and the relationship between neighbouring ski areas are investigated. Data for the analysis cover the 32 largest ski resorts and the winter seasons 1998/1999 to 2018/2019, linked to information on natural snow depth from the nearest weather station (sources: SLAO and SMHI). Official data on snow depth are used to guarantee consistency in quality and measurement over the whole time period studied.

Many studies examine the relationship between snow depth and the number of skier visits or lift ticket sales using econometric models (Hamilton, Brown and Keim 2007; Shih, Nicholls and Holecek 2009; Gonseth 2013, Damm, Köberl and Prettenthaler 2014; Demiroglu et al. 2015; Holmgren and McCracken 2014, Malasevska et al. 2017, Steiger et al. 2019 for a review of the literature) although none of them consider the possible neighbourhood impacts.

The number of skiers might not only depend on natural snow depth in the ski area itself, but also on the snow depth in neighbouring locations. In the literature on ski tourism, this behaviour is commonly referred to as spatial substitution (Dawson, Havitz and Scott 2011; Cocolas et al. 2016). Ski resorts are often located close to each other, so the number of skier visits might depend on the performance of their neighbours.

The main contribution of this study is the identification of spatial effects and the dynamic panel data modelling of the demand for downhill skiing. This means that factors of the neighbourhood are considered as well as the persistence in skier visits. While static spatial panel data models are increasingly popular to use in empirical analysis of tourism demand (Yang and Zhang, 2019), this is the first dynamic application where also cross-sectional dependence is controlled for (Ciccarelli and Elhorst, 2018). A panel data model also controls for presumptive measurement errors that are time invariant, such as distances between the weather stations and the reference points at the ski resorts.

## Conceptual background and previous literature

Several studies investigate the role of climate factors and weather variability in winter tourism. Most studies use data on overnight stays or arrivals at the village or regional level for a single country (Falk 2010; Töglhofer et al. 2011) or comparable data on tourism flows at the regional level for several countries (Damm et al. 2017; Falk and Lin 2021). Damm et al. (2017) provide estimates on the snow sensitivity of overnight stays in the winter season based on NUTS3 data from twelve European countries in the period 2005 to 2010. In 66 out of 119 of these regions, there is a significantly positive relationship (at the ten per cent level) between the measure of snow conditions and overnight stays. In Sweden, there is a significant correlation (at the 5 per cent level) in three out of five regions: Värmland, Dalarna and Jämtland, while only a weak association can be found for the regions Västerbotten and Norrbotten, which are both located at a higher latitude (Damm et al. 2017).

Another important finding of the empirical studies is that the magnitude of the relationship between natural snow depth and winter overnight stays or arrivals in mountain destinations is small and decreases over time. This finding is valid not only for the Scandinavian mountains (Falk and Lin 2021; Töglhofer et al. 2011) but also for the European Alps, possibly with the exception of resorts at low or medium altitudes where there still is a link (Töglhofer et al. 2011). There are also a number of recent studies that investigate the relationship between snow conditions and skier visits using econometric methods (Pickering 2011; Gonseth 2013) or ski lift revenues (Shih et al. 2009; Falk and Hagsten 2016; Falk and Lin 2018).

Revenue from ski lift tickets or skier visits is likely to be more sensitive to fluctuations in snow conditions than tourism indicators such as overnight stays or arrivals at winter resorts, as the former also includes turnover from day visitors. The latter group is particularly flexible and responsive to adverse snow conditions (Damm et al. 2017).

Other studies use the survival of ski resorts in New England as measures of performance (Beaudin and Huang 2014) and find that reduced snowfall directly contributes to their closure. While previous studies show that natural snow depth is crucial for downhill skiers, recent research reveals that the importance of natural snow is diminishing (Falk and Lin 2018, 2021). One explanation behind this is the increase in investments in snowmaking equipment. The importance of snowmaking is confirmed by Gonseth (2013), who demonstrates that the sensitivity of skier visits in relation to snow depth depends on the snowmaking capacity of Swiss ski resorts.

Research based on daily data is also common in the field and includes Hamilton et al. (2007), Shih et al. (2009), Damm et al. (2014), Demiroglu et al. (2015) and Malasevska et al. (2017). These analyses typically lead to significant and large estimates of the impact of natural snow and other weather conditions on skier visits since skiing is a weather-dependent sport. High temperatures affect the quality of the snow, while fresh snowfall makes for beautiful scenery and often allows off-piste skiing.

Yet another strand of research compares extreme winter periods with a lack of snow with those in climatically normal seasons. Rutty et al. (2017), for instance, note that skier visitation declines by 10 per cent during the record warm winter of 2011–2012 in Ontario (Eastern Canada), characterised by a 20 per cent overall decline in natural snowfall compared to a climatically normal season. A parallel approach is used on the number of passengers transported uphill for the extreme winter season 2006/2007 in Austria, with similar results (Steiger 2011). Pickering (2011) shows that a lack of natural snow leads to a strong decline in skier visits, based on data for the entire winter season of Australian ski resorts. According to Dawson et al. (2009) and Steiger (2011), who use data from ski lift operators, the negative demand effects due to the lack of snow or exceptional winter temperatures are smaller in more recent than in earlier periods.

In general, available studies can be differentiated according to the frequency of the data (daily, monthly or annual data). However, when analysing the relationship between snow depth and skier demand, data for the entire winter season and over a longer period of time are more appropriate. Martín (2005) points out that the frequency of the data is crucial in estimating the relationship between tourism and weather. Over a longer period of time (e.g. a whole winter season), weather tends to have less of an impact on tourism flows, as visitors can change to periods of good snow conditions (intertemporal substitution). Besides this, there is also a possibility to relocate to another ski area nearby with better snow conditions. The closer the ski areas are to each other, the more likely it is that they have similar snow conditions.

Another feature of the literature is that the methods are diverse, ranging from descriptive statistics (Pickering 2011) to econometric modelling (Hamilton et al. 2007) and stated choice references (Steiger et al. 2020). While dynamic econometric models are standard, including both time series models (e.g. Hamilton et al. 2007; Falk and Lin 2018) and panel data models (Falk 2010; Töglhofer et al. 2011), spatial econometric models are rarely used in the field of demand for downhill skiing. Overall, the studies are also complicated to compare because of variations in the length of the period studied, the level of aggregation and the measurement of demand and visitor flows. In summary, few studies consider the role of snow conditions in neighbouring ski areas.

There are several reasons to believe that the strength of the relationship between natural snow variability and demand for downhill skiing is low. In response to climate change, resorts worldwide invest heavily in snowmaking and other adaptation measures (snow management and storage, etc.) (Steiger and Scott 2020). In Sweden, the installation of snowmaking systems started in the ski resort of Lindvallen/Sälen in 1977 (Skistar annual report 1999/2000). Already towards the end of the 1990s, investment in snowmaking systems appears a top priority for the leading ski lift operator in Sweden (Skistar annual report 2000, p. 7). Snowmaking at the three ski resorts belonging to the Skistar group (Åre, Sälen and Vemdalen) increased from 50 per cent in the winter season 2003/2004 to 72 per cent in 2019/2020 (Table A1, Appendix). Certain ski resorts, especially those with large resources, are thus becoming largely independent of natural snow as long as temperatures are cold enough to produce it. There is even a possibility that the relationship between natural snow depth and skier visits may turn out to be negative. Such effects could occur in snow rich winters when skiing is possible also in the southern, densely populated parts of Scandinavia, offered by smaller resorts closer to the home (Skistar 2018/2019).

In Sweden, both downhill and cross-country skiing are popular, where the latter is typically undertaken as a one-day (or part of the day) trip (Fredman and Heberlein 2003). Loomis and Crespi (1999) compare different snow-based winter sports and find that the number of visitor days in cross-country skiing areas is among the most sensitive to global warming. Snowmaking on cross-country tracks is generally less widespread. There are about 1500 registered cross-country tracks providers, 140 of which are equipped with snowmaking facilities (source: www.skidspar.se). Even without snow, a cold winter season may be attractive for other sports such as long-distance ice skating, which is popular to undertake on lakes and the sea when the weather conditions so allow. These activities are less formally organised and generally not commercial but there are networks that local clubs can join.^2^

Thus, there are several aspects that may reverse the relationship between natural snow depth and skier visits. Visitors may also adapt to winter seasons with poor natural snow conditions. In this case, the impact of variations in snow depth on skier demand is small. Based on literature, the demand for downhill skiing seems to become less dependent on natural snow due to technological progress and other adaptation measures undertaken by the providers. This leads to the derivation of the first hypothesis:

- H1: The relationship between demand for downhill skiing and natural snow conditions is changing pattern.

Increased demand for downhill skiing may appear when there is a poor coverage of natural snow, since there is a group of operators that guarantee snow-secure slopes, while other snow-dependent winter sports (cross-country skiing) are more dependent on natural snow. This is referred to in the literature as activity-based substitution (Dawson et al. 2011) and leads to the formulation of the second hypothesis:

- H2: The demand for downhill skiing increases when the coverage of natural snow is generally poor.

The literature on the substitutability of leisure activities distinguishes between substitution of activities and spatial substitution (location) (Dawson et al. 2011; Cocolas et al. 2016). This means that a negative correlation between snow depth and skier visits could reflect a substitution relationship between different winter sports areas. Because of this, it is important to account for the influence of the snow depth in neighbouring ski areas. Such types of spatial interdependencies are neglected in literature, except in a study by Hamilton et al. (2007), who show that demand for downhill skiing is more dependent on snow conditions in nearby towns than in the ski areas themselves. This leads to the third hypothesis:

- H3: There is a relationship between demand for downhill skiing and the snow depth in the neighbouring ski areas.

Another aspect of importance is whether the ski resorts collaborate or if they are competitors. In the European Alps, for instance, the resorts are often located close to each other, resulting both in a competitive market for skiers and opportunities to collaborate on joint ski lift passes. However, this situation applies to a lesser extent to Sweden, where there are only a few agglomerations of ski resorts, mainly in the counties of Dalarna and Jämtland. Thus, neighbouring ski areas can be a threat to performance but may also complement each other. Several studies find that the number of visitors (or tourists) at a given location is positively related to the number of visitors at the neighbouring locations (Yang and Wong 2012). This can be interpreted as a significant spillover effect between locations, and not taking these effects into account leads to omitted variable bias.

Empirical studies show that spillover effects are likely to be important. Holmgren and McCracken (2014) exhibit that certain types of skiers are more likely to visit another nearby area if the competitor lowers its prices. This suggests that ski areas are substitutes. If ski areas cooperate with each other by offering joint ski passes, the performance of nearby ski resorts goes hand in hand, leading to the fourth hypothesis:

- H4: There is a relationship between the demand for downhill skiing in one resort and the number of visitors in the neighbouring area.

Several studies show that past tourism or visitor flows are a significant and important predictor of current flows, and thus the degree of persistence gives an indication of how loyal tourists are to a particular destination (Peng et al. 2015). The aspect that this is also valid for ski tourism is considered in the fifth hypothesis:

- H5: There is persistence in the demand for downhill skiing.

The five hypotheses formulated will be tested using a dynamic spatial panel data model.

## Empirical model

According to theory, demand for downhill skiing depends on prices, income and possibly certain additional factors (Englin and Moeltner 2004; Falk and Hagsten 2016). These variables are generally not available at the ski resort level and are instead captured by the time dummy variables in the application on demand for downhill skiing. Given this, the dynamic spatial panel fixed-effect model is specified as follows:

1 $$l{v}_{t}=\tau l{v}_{t-1}+{\rho }_{1}Wl{v}_{t}+\beta l{s}_{t}+{\rho }_{2}Wl{s}_{t}+\Gamma {C}_{t}+a+{u}_{t}$$

where the dependent variable is *lv,* an *N*-dimension vector containing the logarithm of the number of skier visits in *N* ski resorts in time *t* (1998/1999 to 2018/2019). The main independent variable is *ls,* the *N*-dimension vector of the logarithm of snow depth, *W is* the spatial weighting matrix, vector $a$ is the fixed effect intercepts for the ski resorts, and *u* is the normally distributed error term. By following Ciccarelli and Elhorst (2018), ${C}_{t}$ is the common factor(s) that controls the possible cross-sectional dependence (CSD). The potential candidates of ${C}_{t}$ include time dummies, cross-sectional means of ${\overline{lv} }_{t}$, ${\overline{lv} }_{t-1}$, ${\overline{ls} }_{t}$ and their combinations. This model contains both spatially lagged and time lagged dependent variables, *lv*. As pointed out by Baltagi (2021), the model corrects for both time-invariant effects, via the spatial lag, and spatial-invariant effects, through the time lag. Omitting any of these effects would lead to a bias in the estimations.

Thus, Equation (1) can be solved as:

2 $$l{v}_{t}={\left(I-{\rho }_{1}W\right)}^{-1}\tau l{v}_{t-1}+{\left(I-{\rho }_{1}W\right)}^{-1}\left(\beta I+{\rho }_{2}W\right)l{s}_{t}+{\left(I-{\rho }_{1}W\right)}^{-1}\left(\Gamma {C}_{t}+a+{u}_{t}\right),$$

where the short-run effects are

3 $$\frac{\delta lv}{\delta ls}={\left(I-{\rho }_{1}W\right)}^{-1}\left(\beta I+{\rho }_{2}W\right),$$

and the long-run effects are obtained by $l{v}_{t}=l{v}_{t-1}$

4 $$\frac{\delta lv}{\delta ls}={\left((1-\tau )I-{\rho }_{1}W\right)}^{-1}\left(\beta I+{\rho }_{2}W\right).$$

The direct effects are defined as the diagonal elements of the right-hand expressions in Equations (3) and (4), while the indirect (spatial) effects appear as the row sums of the non-diagonal elements. When ${\rho }_{1}$ and ${\rho }_{2}$ are both zeros, there are no spatial effects.

Model (1) is referred to as the dynamic spatial Durbin model (dynamic SDM) which includes the spatial lagged independent variable, $Wl{s}_{t}$, (Elhorst 2014) and also nests the dynamic spatial autoregressive (dynamic SAR) model when ${\rho }_{2}=0$, which excludes this exogenous spatial effect. The more general SDM model also includes the interaction of the spatially and temporally lagged dependent variable, $Wl{v}_{t-1}$ (Debarsy, Ertur and LeSage 2012). However, this term is never significant in the estimations and is thus ignored in the present study. In the dynamic SAR model, the spatial effects, according to (3) and (4), are not due to the local spatial spillover effects associated with ${\rho }_{2}W$. The spatial effects are mainly due to endogenous spatial effects, which are referred to as global spillover effects. As the name suggests, these effects are independent of the distances between the ski resorts (Elhorst 2012).

The model selection is based on the hypothesis test of ${\rho }_{2}=0$ from estimating equation (1). There are additional criteria such as the CSD (cross-sectional dependence) test; stationarity of residuals as well other model selection criteria including AIC, SIC, log-pseudolikelihood and overall R^2^. Ciccarelli and Elhorst (2018) also point out the stability condition. However, this condition depends on the weighting matrix W chosen. Because of this, this study refrains from verifying this condition.

The estimation of the fixed effects model (1) is carried out by the bias-corrected quasi-maximum likelihood (QML) for spatial panel data (Yu, de Jong and Lee 2008). This estimator requires that the panel data are strongly balanced and thus do not contain any missing values (Belotti, Hughes and Mortari 2017). Using a balanced panel of large ski lift operators leads to a selected sample and causes the so-called survivor bias as described for manufacturing and service firms in general (Delmar and Wiklund 2008). Focusing on survivors and medium-sized and large ski lift operators might lead to an underestimation of the relationship between natural snow depth and skier visits.

## Data

Data originate from two main sources: Skier visits from the Swedish Ski Lift Operators Association (SLAO) and snow depth from the Swedish Meteorological Service (SMHI). Skier visits in the winter season are defined as one person visiting a ski area during the whole or part of one day for downhill skiing or snowboarding. Seasonal card holders are automatically considered to ski 21 days (SLAO 2019). Information on snow depth at the daily level is obtained from 47 weather stations near the ski resorts (Table A2, Online Appendix). These weather stations are mainly linked to the valley stations. As the difference in altitude between the mountain and valley stations is small compared to the European Alps, there are good reasons to believe that the snow conditions at the valley stations are reasonable approximations of the conditions in the ski area. Average snow depth in metres is calculated as the mean between December 15 and March 31 based on daily data. These data are matched to the nearest ski area using longitude and latitude (Table A2, Online Appendix).

Data are available for the 50 largest ski areas. According to the Swedish Ski Association, there are currently around 200 members compared to 230 during the 2008/2009 winter season (SLAO, 2009, 2019). Linking the data over time results in a balanced panel dataset with 32 ski operators (Table A3, Appendix). These ski resorts account for about 82 per cent of total skier visits (winter season 2018/2019) (SLAO 2019). The spatial weighting matrix constructed, W (32*32 matrix,) uses the inverse of the geographical distances in kilometres between the ski resorts. Information for small ski resorts with less than 20,000 skier visits is generally not available.

**Fig. 1:**
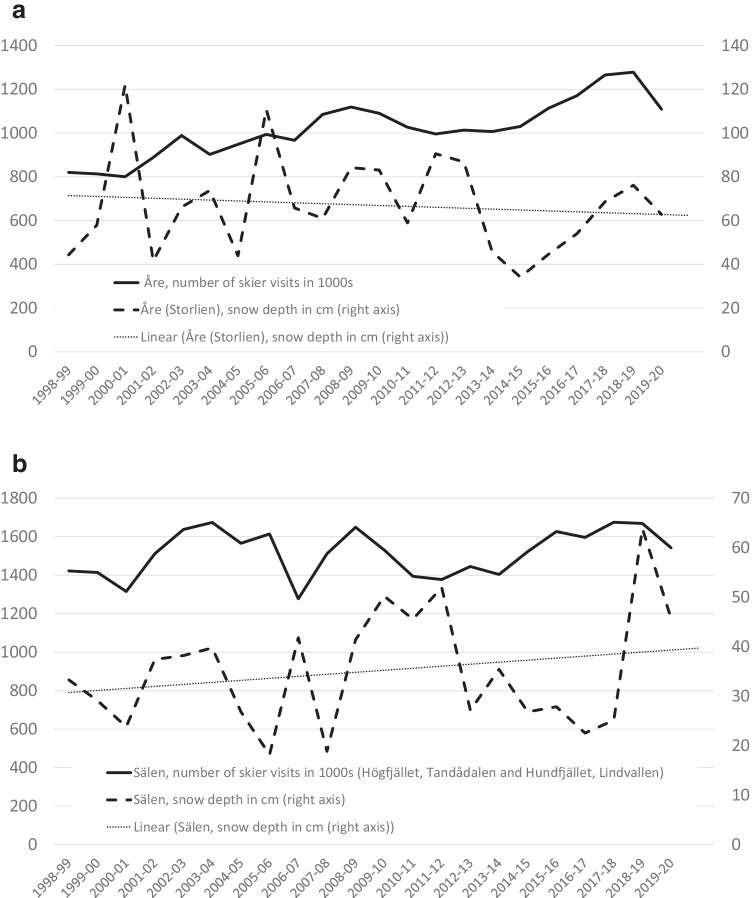
A and B Evolution of snow depth and number of skier visits. Source: SMHI and SLAO

Wikipedia lists 259 ski resorts in Sweden, but more than 60 are no longer in operation (25 percent).^3^ Common reasons behind the disappearance are closure of the ski area and sale of the lifts, bankruptcy, lack of snow, conversion to mountain bike park or lack of new owners. A majority of the closed ski areas are located in the south of Sweden: Stockholm (14), Småland (5), Västergötland (4) and all the others (Bohuslän, Halland, Skåne, Värmland, Västmanland) 3 each. Ski resorts in the north of Sweden (Jämtland, Lapland, Västerbotten, Dalarna, Hälsingland, Härjedalen, Medelpad and Norrbotten), on the other hand, have fewer closures (12 in total). This indicates that climate zones and snow availability may lie behind closures (Rice et al. 2021). The average number of skier visits per season is 204,000 (Table 1). Sälen in Dalarna County receives the highest number of skier visits (1.67 million visitors in the winter season 2018/2019). The average snow depth is 0.43 metres and Kittelfjäll has the largest snow cover of 1.38 metres in the winter season 2018/2019. At the other end of the scale is Vallåsen in the southern Sweden with 0.002 metres of snow in the winter season 2017/2018. Åre has a downward trend in natural snow depth, while the opposite is valid for Sälen (Fig. 1A). Despite this, both resorts encounter and increase number of skier visits Fig. 1B).

**Table 1:** Descriptive statistics 1998/1999 to 2018/2019

	Visitors in 1000s	Snow depth in metres
Mean	203.9	0.43
Std. Dev.	310.2	0.27
Maximum	1675.4	1.39
Minimum	1.0	0.003
	ln(visitors)	ln(snow)
Mean	4.6306	-1.1273
Std. Dev.	1.0734	0.9159
Maximum	7.4238	0.3257
Minimum	0.0000	-5.8748
CSD test	27.50***	26.13***
CADF (0)	-0.77	-15.19***
CADF (1)	4.12	-5.88***
CADF (2)	4.08	-0.59

Asterisks ***, ** and * denote significance at the 1, 5 and 10 per cent levels. CSD indicates the statistics of the cross-sectional dependence test with a null hypothesis of cross-sectional independence (Pesaran, 2021). CADF reports the statistics of second-generation CPIS panel unit-root test (Pesaran, 2007). The numbers in parentheses in the context of CADF indicate lags. The CADF test for ln(visitors) is subject to a trend. The CADF test for ln(snow) is not subject to a trend. The test results for ln(visitors) are consistent: the unit root is not rejected. The conclusion for ln(snow) is mixed. The null variant is rejected up to lag 1 but not at two lags

## Empirical results

Estimation results of the dynamic spatial Durbin model and the spatial autoregressive model show that the long-run impact of natural snow on skier visits is only partly significant and when they are, there is an adverse relationship, which means that Hypothesis 1 cannot be rejected (Tables 2 and 3). There is also a significant negative relationship between the natural snow depth in the ski area and the number of skier visits in the short term. The short-run elasticity of skier visits related to snow depth is -0.07 and is significant at the one per cent level (Table 3). This means that a decrease in natural snow depth by 10 per cent leads to a 0.7 per cent increase in the number of skier visits. Hypothesis 2 is thus fulfilled.

**Table 2:** Results of the SDM estimations

	SDM 1	SDM 2	SDM 3
ln(visitors)(-1)	0.527*** (0.11)	0.527*** (0.11)	0.518*** (0.12)
ln(snow)	-0.060** (0.03)	-0.060* (0.03)	-0.062** (0.03)
W			
ln(visitors)	0.343*** (0.07)	0.342*** (0.07)	0.348*** (0.05)
ln(snow)	-0.031 (0.05)	-0.040 (0.05)	-0.006 (0.04)
Short run			
Direct	-0.064** (0.03)	-0.064** (0.03)	-0.065** (0.03)
Indirect	-0.074 (0.06)	-0.089 (0.07)	-0.041 (0.05)
Total	-0.138*** (0.05)	-0.153*** (0.05)	-0.106** (0.05)
Long run			
Direct	-0.161 (0.15)	-0.163 (0.16)	-0.152*** (0.06)
Indirect	-0.679 (4.23)	-0.740 (4.75)	-0.440 (0.62)
Total	-0.839 (4.36)	-0.903 (4.89)	-0.592 (0.63)
Time dummies	year07, year14, year18		
Common factor	no	m(ln(snow))	m(ln(visitors))(-1)
CSD test	1.545 [0.12]	1.625 [0.10]	1.632 [0.10]
CADF test	387.05***(0) 168.55***(1) 122.60***(2)	385.96***(0) 169.46***(1) 123.17***(2)	366.72***(0) 165.33***(1) 117.24***(2)
AIC/BIC	227/263	229/270	204/244
Log-pseudolikelihood	-108	-108	-96
Overall R^2^	0.87	0.87	0.88

Asterisks ***, ** and * denote significance at the 1, 5 and 10 per cent levels. The standard errors are reported within the parentheses. The estimations are based on robust standard errors and conducted using the Stata command *xsmle* (Belotti et al. 2017) that implements the bias-corrected QML method for spatial panel data (Yu et al. 2008). Statistics and p values in square brackets are reported for stability tests. CSD indicates the statistics of the Pesaran (2021) cross-regional dependence test on the residuals and its corresponding p value is in square brackets. CADF reports the statistics of panel unit root test where the CADF statistics is based on the MW (Maddala and Wu 1999) panel unit root test without trend. The numbers in the parentheses associated with these statistics indicate lags. The variable m(ln(snow)) is the cross-sectional mean of log of snow depth. Time dummies used here are the ones representing the years 2007, 2014 and 2018

**Table 3:** Results of the SAR estimations

	SAR 1	SAR 2	SAR 3
ln(visitors)(-1)	0.527*** (0.11)	0.528*** (0.11)	0.519*** (0.12)
ln(snow)	-0.072*** (0.02)	-0.066*** (0.03)	-0.065*** (0.02)
W			
ln(visitors)	0.340*** (0.07)	0.342*** (0.07)	0.349*** (0.05)
Short run			
Direct	-0.074*** (0.02)	-0.069*** (0.03)	-0.067*** (0.02)
Indirect	-0.036*** (0.01)	-0.033** (0.01)	-0.034*** (0.01)
Total	-0.110*** (0.03)	-0.102*** (0.04)	-0.101*** (0.03)
Long run			
Direct	-0.169 (0.16)	-0.176 (0.17)	-0.159** (0.07)
Indirect	-0.278 (4.82)	0.849 (5.15)	-0.547 (1.66)
Total	-0.447 (4.97)	-1.025 (5.32)	-0.706 (1.72)
Time dummies	year07, year14, year18		
Common factor	no	m(ln(snow))	m(ln(visitors))(-1)
CSD test	1.682* [0.09]	1.412 [0.16]	1.653* [0.10]
CADF test	385.12***(0) 170.24***(1) 122.42***(2)	387.90***(0) 167.58***(1) 121.38***(2)	367.65***(0) 165.40***(1) 117.38***(2)
AIC/BIC	226/257	227/263	202/238
Log-pseudolikelihood	-108	-108	-96
Overall R^2^	0.87	0.88	0.88

Asterisks ***, ** and * denote significance at the 1, 5 and 10 per cent levels. The standard errors are reported within the parentheses. See Table 2 for further information

However, the long-run elasticity is only partly significant and depends on the specification (Table 3, SAR 2 and SAR 3). There is no relationship between demand for downhill skiing and snow depth in the neighbouring areas, although a spatial effect is found for the number of skier visits implying that Hypothesis 3 cannot be confirmed. The weighted number of visitors to the neighbouring ski areas is significantly correlated with a coefficient of 0.35 at the one per cent level (Table 3, SAR 2 and SAR 3). This suggests that the neighbours complement rather than substitute each other, implying that Hypothesis 4 cannot be rejected. In contrast to natural snow, the number of skier visits is highly persistent as indicated by the coefficient of the lagged dependent variable τ. This coefficient has a magnitude of 0.52 and is significant at the five per cent level (Table 3. SASR 3). Thus, the fifth hypothesis can also not be rejected at conventional significance levels.

The next step is to report the estimation results in detail. Results obtained from the dynamic spatial Durbin model estimations (dynamic SDM) (1) show that the spatially weighted level of snow depth is not significant (${\rho }_{2}=0$ in Table 2), indicating that local spillover effects of snow depth are not relevant. Therefore, the spatial effects, if any, would be mainly due to global spatial effects, through the number of skier visits and irrelevant to distances between the resorts. These results also lead to the conclusion that the SAR models are more appropriate (Table 3). There are three SAR models considered where the difference lies in the common factors adopted. The first model, SAR1, contains no common factors as suggested by Ciccarelli and Elhorst (2018), but three time-dummy variables for 2007, 2014 and 2018. The presence of cross-sectional independent residuals cannot be rejected at the five per cent level and the panel unit-root tests (Maddala and Wu 1999) suggest stationary residuals. The second and the third models adopt the common factors, cross-sectional averages of the logarithm snow depth and cross-sectional averages of the lagged logarithm of skiing visitors besides the time dummies. Given the similar simultaneous testing results of cross-sectional independence and stationary residuals, AIC, BIC, log-pseudolikelihood and overall R squared would suggest that the final selection would be the model SAR3. Nevertheless, two other models perform well. They, therefore, can be regarded as robustness checks. All results from the three models are consistent.

Overall, the results show that the impact of natural snow on skier visits is only partly significant and if so there is an adverse relationship. The results are largely consistent with Falk and Lin (2021) for Jämtland and Dalarna and are based on aggregate ski lift revenues for Sweden (Falk and Lin 2018). Töglhofer et al. (2011) also demonstrate an insignificant relationship between snow depth and overnight stays in ski resorts for high altitude areas (>1800 m) in Austria. However, the findings contradict previous studies using earlier data. Falk and Hagsten (2016) find that snow depth matters in the early season December to January. Explanations for the differences are related to variations in research design, levels of aggregation and time windows for the analyses. Studies based on lower frequency data might lead to higher relationships because they cannot account for the temporal substitution effect as explained by Dawson et al. (2011). An example is that a bad winter start can be compensated by a good peak season. The insignificant impact of natural snow on demand in ski resorts can also be explained by advances in snowmaking. Without natural snow, cold weather and modern equipment are enough to put the slopes in good condition for skiing. The result that natural snow depth in the ski area is independent of the snow conditions in neighbouring areas is a new finding in the literature, emphasising the changed role of ski lift operators that not only includes grooming of the slopes but also large production of snow.

In the short run, the direct effects of snow depth are negative and highly significant. This indicates that the number of visitors increases when the ski area has less natural snow. One explanation behind this impact is that a general lack of winter climate in the ski area leads to an increase in downhill skiing activities, since other activities such as cross-country skiing or long distance ice-skating are possibly more difficult to undertake.

In times of good snow coverage, there might also be a boom for the smaller ski resorts less equipped with snowmaking facilities. Smaller ski areas are mainly located in the south of Sweden, not seldom close to cities (Demiroglu et al. 2019). When there is a lack of snow, the need to utilise the larger ski resorts with advanced snowmaking facilities is larger. Unfortunately, this type of spatial substitution effect cannot be tested in this study due to data deficits. There is also a large degree of volatility among the smaller resorts, as several of them have left the market and thus cannot be included in an empirical analysis that requires a balanced panel of observations. Nevertheless, the substitution effect is unlikely to be large, since the smaller resorts account for less than 20 per cent of the total number of skier visits (SLAO 2019). Overall, the results show that economic effects such as persistent visitor behaviour and strong performance of neighbouring ski areas are important, while fluctuations in natural snow depth are not.

Several robustness checks are carried out. One of them uses an interaction term between latitude (< latitude 60, which corresponds to the line south of Falun) and snow depth to test whether ski resorts in the south of Sweden are more affected by snow variability. Demiroglu et al. (2020) point out that in Scandinavia, not only altitude but also high latitude is a comparative advantage in times of global warming. Unreported results show that the interaction term is not significant. Besides latitude, altitude and maritime weather conditions could influence the relationship between performance and snow depth. However, the sample size of 32 ski areas is too small to allow separate regressions or interaction terms for these types of ski areas. In addition, other definitions of average snow depth are used such as two alternative periods: (i) 1 December to 31 March and (ii) 15 December to 15 April. Unreported results reveal that the relationships between average snow depth and skier visits are not sensitive to these variations of definitions.

A final robustness check relates to investments in and installations of new ski lifts. In the sample period, there are several larger lift installations such as new gondolas that could affect visitations (see Demiroglu et al. 2019). Åre invested heavily in the run-up to the recent World Championships. However, the inclusion of the investments is beyond the scope of the study as these new investments do not lead to an increase in the terrain in the last 15 years (Skistar, annual reports).

## Discussion and conclusions

This study investigates how natural snow depth affects the number of skier visits. The main novelty of the study is the inclusion of spatial dependencies and persistence, in terms of both snow depth and the number of visitors. Evidence is provided for the 32 largest ski resorts in Sweden during the period 1998/1999 to 2018/2019. Results of the spatial estimations show that snow depth in neighbouring ski areas as well as in the ski area itself have no influence on the number of skier visits in the long run. In the short run, there is a negative relationship between snow depth and skier visits.

One explanation for the adverse effects is that a lack of snow in the ski area leads to an increase in downhill skiing because ski resorts offer ski-runs covered by snowmaking to a much larger extent than many other alternative winter sports activities such as cross-country skiing, long-distance ice skating or snowmobiling. The three largest ski resorts in Sweden have a snowmaking capacity for 72 per cent of the slopes in 2018/2019 as compared to 50 per cent in earlier years (2003/2004). Another explanation is that skiers are moving from smaller ski resorts, which are less often equipped with modern snowmaking systems, to larger ones. Unlike snow conditions, economic and regional factors such as a high persistence in skier visits and a high positive dependence on skier visits in neighbouring ski areas are of marked importance for the performance of ski resorts. This suggests that neighbouring ski areas complement rather than substitute each other.

The findings lead to several conclusions. Lack of snow depth is not relevant for the performance of the 32 ski resorts in the long run in the period covered by the sample. This is remarkable since this period is already affected by global warming. The results also reflect both a successful adjustment by the ski resorts, but also that there are possible spatial substitution effects that cannot be measured in this study. These changes are visible in the short term, where adverse effects are observed related to the fact that more natural snow leads to a shift away from the larger resorts, possibly to smaller ones and other winter sport activities.

There are several limitations that should be noted. Small ski lift operators are not considered due to a lack of data. The use of a balanced panel of large and surviving ski lift operators results in a highly selected sample. Focusing on large and surviving firms could lead to an underestimation of the relationship between natural snow depth and skier visits. In addition, the effects of ski lift prices and cyclical factors could not be taken into account due to similar reasons.

Another limitation is that geographical distance is used as the spatial weight matrix. The average travel time is likely to decrease in the future (due to new airports and better roads) and ski resorts will become more accessible from larger cities. This means that future studies should include travel time and a direct measure of improved transport accessibility in the model. Other ideas include the use of a shorter time window for the analysis to allow more detailed snow depth data sourced directly from the ski resorts themselves, relating to both natural and fabricated snow.

Additional research ideas encompass a broader set of weather indicators such as temperatures and daylight hours, or to jointly model the probability of survival of ski areas and performance measured as skier visits. However, such an exercise requires new data. It would also be possible to expand the analysis to include the spatially lagged number of visitors or to apply the methodology to other ski areas in the world.

### Supplementary Information

Below is the link to the electronic supplementary material.

Supplementary file1 (DOCX 45 KB)

## Footnotes

1 Source: SMHI based on the climate indicator number of frost days www.smhi.se/klimat, accessed 2021, October 22.

2 Source: https://www.skridsko.net/skridskonet/artikel/?artikelID=61991 (accessed, 2021, November 12).

3 This paragraph is based on following source: Lista över skidanläggningar i Sverige – Wikipedia , List of ski resorts in Sweden, https://sv.wikipedia.org/wiki/Lista_%C3%B6ver_skidanl%C3%A4ggningar_i_Sverige (accessed, 2021, October 2021).
