# Supplementary material for: Spatial influence on the distribution of downhill skiers in Sweden
Source: Int J Biometeorol. 2022 Mar 31;68(3):535–45. doi: 10.1007/s00484-022-02259-5 (PMC10864428; doi:10.1007/s00484-022-02259-5)
Supplement: Supplementary file 1 — Supplementary file1 (DOCX 45 KB) [file 484_2022_2259_MOESM1_ESM.docx]

Online Appendix

*Table A1. Snow making capacity of selected Swedish ski resorts*

|  | **Åre** | | |
| --- | --- | --- | --- |
| Winter season | Total area of groomed slopes | Area covered by snowmaking systems | Proportion of snowmaking systems |
|  | square metres | square metres | per cent |
| 2003/2004 | 4,370,000 | 1,920,000 | 44 |
| 2004/2005 | 4,400,000 | 2,000,000 | 45 |
| 2007/2008 | 4,500,000 | 2,220,000 | 49 |
| 2009/2010 | 4,500,000 | 2,220,000 | 49 |
| 2012/2013 | 3,204,478 | 2,352,760 | 73 |
| 2014/2015 | 3,253,394 | 1,958,590 | 60 |
| 2015/2016 | 3,253,394 | 1,958,590 | 60 |
| 2018/2019 | 3,253,394 | 1,958,590 | 60 |
| 2019/2020 | 3,304,194 | 2,058,405 | 62 |
|  | **Sälen** | | |
|  | Total area of groomed slopes | Area covered by snowmaking systems | Proportion of snowmaking systems |
|  | square metres | square metres | per cent |
| 2003/2004 | 3,600,000 | 1,850,000 | 51 |
| 2004/2005 | 3,600,000 | 1,850,000 | 51 |
| 2007/2008 | 3,135,000 | 1,900,000 | 61 |
| 2009/2010 | 3,200,000 | 1,850,000 | 58 |
| 2012/2013 | 2,877,000 | 1,850,000 | 64 |
| 2014/2015 | 2,882,000 | 2,215,000 | 77 |
| 2015/2016 | 2,882,000 | 2,215,000 | 77 |
| 2018/2019 | 2,890,500 | 2,259,000 | 78 |
| 2019/2020 | 2,890,500 | 2,291,000 | 79 |
|  | **Vemdalen** | | |
|  | Total area of groomed slopes | Area covered by snowmaking systems | Proportion of snowmaking systems |
|  | square metres | square metres | per cent |
| 2003/2004 | 1,230,000 | 664,000 | 54 |
| 2004/2005 | 1,380,000 | 950,000 | 69 |
| 2007/2008 | 1,360,000 | 980,000 | 72 |
| 2009/2010 | 1,390,000 | 1,003,800 | 72 |
| 2012/2013 | 1,606,414 | 1,283,082 | 80 |
| 2014/2015 | 1,646,244 | 1,329,782 | 81 |
| 2015/2016 | 2,013,900 | 1,502,853 | 75 |
| 2018/2019 | 2,013,900 | 1,502,853 | 75 |
| 2019/2020 | 2,013,900 | 1,502,853 | 75 |

Source: Ski star annual reports, various issues.

*Table A2. List of the weather stations used to calculate snow depth*

| Ski resort | Weather station | Latitude | longitude | Altitude in metres |
| --- | --- | --- | --- | --- |
| Åre | Storlien | 63.28 | 12.12 | 583 |
| Björkliden | Abisko | 68.35 | 18.82 | 392 |
| Bjursås | Falun | 60.62 | 15.66 | 161 |
| Branäs/Sysslebäck | Kindsjön D | 60.66 | 12.71 | 425 |
| Bydalen/Hallen | Morsil | 63.32 | 13.65 | 420 |
| Bygdsiljum | Brande | 64.33 | 20.96 | 60 |
| Bygdsiljum | Lovanger | 64.41 | 21.29 | 25 |
| Dundret/Gällivare | Malmberget | 67.14 | 20.64 | 359 |
| Fjätervålen/Särna | Särna D | 61.69 | 13.18 | 425 |
| Flottsbro/Huddinge | Norsborg | 59.25 | 17.80 | 9 |
| Flottsbro/Huddinge | Sodertalje | 59.21 | 17.63 | 43 |
| Hamrafjällets/Tänndalen | Malmagen | 62.61 | 12.16 | 785 |
| Hamrafjällets/Tänndalen | Ljusnedal | 62.55 | 12.60 | 585 |
| Hassela | Åsnorrbodarna | 62.15 | 17.17 | 143 |
| Hemavan-Tärnaby | Hemavan-Mosekälla | 65.73 | 15.06 | 465 |
| Hemavan-Tärnaby | Nordanås | 65.46 | 16.09 | 403 |
| Idre Fjäll | Idre D | 61.86 | 12.73 | 455 |
| Isaberg | Hestra | 57.47 | 13.63 | 220 |
| Järvsö | Simeå | 61.57 | 16.33 | 139 |
| Kåbdalis | Puottaure | 66.19 | 20.25 | 320 |
| Kittelfjäll | Kittelfjäll | 65.26 | 15.52 | 553 |
| Kläppen/Sälen | Mångsbodarna | 61.08 | 13.62 | 424 |
| Kungsberget | Kungsberget | 60.77 | 16.47 | 150 |
| Kungsberget | Hillersboda | 60.71 | 15.94 | 135 |
| Lofsdalen | Myskelåsen | 62.33 | 12.65 | 770 |
| Orsa Grönklitt | Orsa | 61.13 | 14.64 | 177 |
| Ramundberget/Funäsdalen | Ljusnedal | 62.55 | 12.60 | 585 |
| Riksgränsen | Katterjåkk | 68.42 | 18.17 | 514 |
| Romme Alpin | Borlänge | 60.49 | 15.43 | 145 |
| Säfsen | Fredriskberg | 60.14 | 14.39 | 305 |
| Sälen (Högfjället, Tandådalen and Hundfjället, Lindvallen) | Nornäs D | 61.43 | 13.23 | 460 |
| Ski Sunne | Hogboda | 59.58 | 13.02 | 92 |
| Storklinten, Boden | Koler | 65.50 | 20.46 | 288 |
| Storlien | Storlien | 63.28 | 12.12 | 583 |
| Stöten/Sälen | Storbron | 61.39 | 12.86 | 540 |
| Valfjället | Charlottenberg | 59.89 | 12.30 | 139 |
| Vallåsen | Örkelljunga | 56.28 | 13.30 | 77 |
| Vemdalen | Ratan | 62.48 | 14.54 | 360 |

Source; SMHI, Swedish Meteorological and Hydrological Institute.

*Table A3. Ski resorts, skier visits and coordinates*

|  | Skier visits in 1000s |  |  |
| --- | --- | --- | --- |
| Ski area | 2018-19 | Latitude | Longitude |
| Åre Skistar | 1278 | 63.399 | 13.081 |
| Bjursås | 70 | 60.767 | 15.462 |
| Branäs/Sysslebäck | 501 | 60.662 | 12.964 |
| Bydalen/Hallen | 135 | 63.091 | 63.091 |
| Bygdsiljum/Bygdsiljum | 27 | 64.357 | 64.357 |
| Dundret/Gällivare | 20 | 67.110 | 67.110 |
| Fjätervålen/Särna | 34 | 61.939 | 12.989 |
| Flottsbro/Huddinge | 27 | 59.244 | 17.876 |
| Hamrafjällets/Tänndalen | 131 | 62.573 | 12.226 |
| Hassela | 70 | 62.077 | 16.726 |
| Hemavan-Tärnaby | 191 | 65.821 | 15.086 |
| Idre Fjäll | 510 | 61.859 | 12.719 |
| Isaberg | 86 | 57.435 | 13.610 |
| Järvsö | 242 | 61.714 | 16.177 |
| Kåbdalis | 79 | 66.157 | 19.986 |
| Kittelfjäll | 44 | 65.255 | 15.487 |
| Kläppen/Sälen | 405 | 61.033 | 13.341 |
| Kungsberget | 293 | 60.806 | 16.627 |
| Lofsdalen | 135 | 62.113 | 13.278 |
| Orsa Grönklitt | 151 | 61.209 | 14.536 |
| Ramundberget/Funäsdalen | 158 | 62.701 | 12.389 |
| Riksgränsen | 30 | 68.354 | 18.817 |
| Romme Alpin | 354 | 60.392 | 15.383 |
| Säfsen | 138 | 60.201 | 14.442 |
| Sälen (Högfjället, Tandådalen and Hundfjället, Lindvallen) | 1669 | 61.169 | 13.259 |
| Ski Sunne | 52 | 59.803 | 13.036 |
| Storklinten, Boden | 31 | 66.021 | 21.249 |
| Storlien | 34 | 63.323 | 63.323 |
| Stöten/Sälen | 338 | 61.275 | 12.899 |
| Valfjället | 21 | 59.826 | 12.256 |
| Vallåsen | 60 | 63.877 | 17.894 |
| Vemdalen | 800 | 62.481 | 13.975 |

Source: SLAO, various issues, google map.
